# Dynamic and Quantitative Method of Analyzing Service Consistency Evolution Based on Extended Hierarchical Finite State Automata

**DOI:** 10.1155/2014/793271

**Published:** 2014-01-08

**Authors:** Linjun Fan, Jun Tang, Yunxiang Ling, Benxian Li

**Affiliations:** ^1^Science and Technology on Information Systems Engineering Laboratory, National University of Defense Technology, Changsha 410073, China; ^2^Department of Telecommunications and Systems Engineering, Universitat Autònoma de Barcelona, 08202 Barcelona, Spain; ^3^Department of Management Science and Engineering, Police Officer College of Chinese Armed Police Force, Chengdu 610213, China

## Abstract

This paper is concerned with the dynamic evolution analysis and quantitative measurement of primary factors that cause service inconsistency in service-oriented distributed simulation applications (SODSA). Traditional methods are mostly qualitative and empirical, and they do not consider the dynamic disturbances among factors in service's evolution behaviors such as producing, publishing, calling, and maintenance. Moreover, SODSA are rapidly evolving in terms of large-scale, reusable, compositional, pervasive, and flexible features, which presents difficulties in the usage of traditional analysis methods. To resolve these problems, a novel dynamic evolution model extended hierarchical service-finite state automata (EHS-FSA) is constructed based on finite state automata (FSA), which formally depict overall changing processes of service consistency states. And also the service consistency evolution algorithms (SCEAs) based on EHS-FSA are developed to quantitatively assess these impact factors. Experimental results show that the *bad reusability* (17.93% on average) is the biggest influential factor, the *noncomposition of atomic services* (13.12%) is the second biggest one, and the *service version's confusion* (1.2%) is the smallest one. Compared with previous qualitative analysis, SCEAs present good effectiveness and feasibility. This research can guide the engineers of service consistency technologies toward obtaining a higher level of consistency in SODSA.

## 1. Introduction

In recent years, service-oriented distributed simulations applications (SODSA) have become a major trend in modeling and simulation (M&S) field [1] mainly because of the enormous popularity of novel information technologies such as service-oriented architecture (SOA) [2], compositional simulation methods [3], cloud computing [4], internet of things [5], gridding, and Web service applications [1, 6, 7]. Owing to the usage of such technologies, SODSA is increasingly featured by service-oriented, composite, large-scale, ubiquitous, and flexible characteristics [8, 9]. For this reason, in order to ensure service consistency, traditional maintenance technologies which do not consider these novel attributes of SODSA exhibit some new difficulties (e.g., bad reusability, troubles of service composition, failures of service encapsulation and messages exchanges). It should be noted, however, that service consistency maintenance plays a critical role in the correctness and reliability of M&S. Therefore, it is crucial for software developers to make use of feasible technologies and methods to maintain service consistency. But prior to this step, we should first analyze the various influential factors that cause service inconsistencies, serving as guidance for the design of service consistency technologies with regard to a specific inconsistent factor. Traditional methods are mostly qualitative and experiential, and they do not consider the dynamic disturbances among factors in service's evolution behaviors such as producing, publishing, calling, and maintenance. Moreover, SODSA are rapidly evolving in terms of large-scale, reusable, composite, pervasive, and flexible features, which presents difficulties in the usage of traditional analysis methods. To solve these problems, the main contributions of this paper are summarized as follows.

- We present a new service consistency evolution model, called extended hierarchical service-finite state automata (EHS-FSA), which is based on FSA theory and considers the dynamic disturbances among main inconsistency factors and simulates all transition behaviors and states of combined consistency attributes sets for each simulation service.
- On the basis of EHS-FSA, we also develop two service consistency evolution algorithms (SCEAs) by running EHS-FSA and then calculating the number of factors occurrences affecting service inconsistency, which provides the quantitative analysis means of impact factors.
- We carry out quantitative evaluation experiments to validate the feasibility and effectiveness of the service evolution model EHS-FSA and the statistical assessment algorithms SCEAs.

As a brief outline of the rest of the paper, Section 2 discusses the background and mechanism of service consistency evolution; Section 3 introduces the service consistency evolution model, including essential formal definitions and EHS-FSA; Section 4 proposes SCEAs and states their main principles; Section 5 gives the quantitative evaluation results and analysis of influencing factors by simulation methods; conclusion and the recommendations for future work are detailed in Section 6.

## 2. Related Work

Over the years, many maintenance techniques have been proposed in prior related studies to maintain all kinds of consistency states and ensure convenient utility of services in service evolution processes from the different emphasis points. Tian et al. [10] consider the software service reuse issues in ubiquitous environments and present a novel reuse approach in service consistency evolution processes, in which the reusable parts of existing services are extracted and directly utilized, and the missing functionalities-based index is then further implemented and built to accelerate the service reuse process. Sindhgatta and Sengupta [11] investigate the model transformation processes and the associated changing service design method in model-based development of SOA and introduce an extensible framework for tracing the dynamic evolution of model and model-based service. Frank and Karl [12] state consistency challenges of service discovery in mobile ad hoc networks and discuss the influencing factors on system efficiency that refers to transmission protocol, correctness of the delivered messages, message overhead, and even geographic distance between service providers and subscribers. Greenfield et al. [13] study the consistency maintenance problems of Web services, ensuring that services calls always be finished in consistent states despite failures and other exceptional events. Reference [13] addresses the relationship between internal service states, messages, and application protocols which facilitate the transformation from the problem of ensuring consistent outcomes into a protocol problem that can be easily validated by established service verification tools. There is also a great deal of other research achievements of service consistency evolution such as the studies in literatures [5, 14–18] that analyse the challenges and troubles and give some significative solutions. However, these solutions are usually adopted in such simulation environments where the number of simulation nodes is fixed and service developments are centralized and inflexible. With the emergence of SODSA, some new traits of software service developments are emerging, leading to traditional analysis methods being less applicable.

As for the above-mentioned former literatures [5, 14–18] that investigate service consistency in traditional distributed simulations compared with SODSA, there exhibit the following deficiencies in analyzing these influencing factors. (1) Only one or some of these factors are discussed, rather than most of enabled influential factors. (2) These studies only explain why these factors lead to service inconsistency, but do not specify the numerical extent which results in such inconsistent phenomena. (3) Dynamic perturbation mechanism between inconsistent factors in service running and their dynamically impact on all possible transitions of service consistency states are disregarded. (4) Existing researches focus mainly on inconsistency factors in special simulation phases or processes and do not synthetically investigate the issues that affect the overall software life cycle. Thanks to some major features of emerging SODSA such as large-scale attributes, dynamic assemblage, redundancy deployment, plug-in procedures, and flexible composition, the simulation nodes in SODSA are transformable, unpredictable, and therefore stochastic. These traits result in an increasingly unreliable and large number of services. Thus, the dynamic evolution mechanism of impact factors on service inconsistency should be exhaustively investigated, and inconsistent details in each simulation service should be carefully monitored in the whole simulation course. By using this analysis way, we can provide an actual and exact picture of the importance of such factors and can enable the excellent design of technologies for maintaining service consistency when it comes to the influencing factors, which facilitate the avoidance of wrong simulation results. When software engineers take into account all influencing factors in such complicated and dynamic simulation environments, they certainly encounter some difficulties in actual analysis, design, and running of service. In this paper, thus, we restrict the analysis to focus on some important inconsistency factors and temporarily ignore secondary ones.

Emerging automata theories [19], graph-based approaches [20], and formal methods [21] in software engineering supply many valuable solutions for describing and analyzing such complicated state changes and interactive behaviors in distributed systems, which provide us with constructive ideas. In particular, a finite state automaton (FSA) [22] has been widely used in the analysis and behavior modeling for various practical moderate-scale systems. However, in large-scale complex distributed systems, as it involves the complexity of sizes, behaviors, states, and properties, a general FSA is not easy to be used to formally describe such systems. The extended hierarchical FSA (EHFSA) [23] that lets a state itself as an FSA and can depict complex state transitions is deepening and an expansion of FSA, avoiding the state space explosion and improving the efficiency of state transition. Considering the multifarious consistency evolution behaviors of atomic and composite services in large-scale SODSA, as well as the layered distribution of service grid, it is feasible that we use EHFSA to describe the consistency state evolution of service behaviors under compositional SODSA. In order to obtain quantitative analysis results, we also design two statistical evaluation algorithms to detect impact factors of service inconsistency.

## 3. Analysis of Service Consistency Evolution

### 3.1. Evolution Mechanism

Considering the actual situations in current SODSA, it is rare that there always exists an appropriate service exactly satisfying the user requirements. Because the popular tendencies of SODSA are that service evolution caters to continually changing requirements and services are dynamically combinated and rapidly updated. Hence, some macrolevel factor refers to service inconsistency, such as the diversity of service behaviors, the complex structures of the service itself, and the service's function overlap, can be observed in service-oriented simulations, which are the focus of service evolution in this research. Based on the above-mentioned factors, we mainly pay attention to the global consistency state evolution of services, monitoring the service state transition triggered by the impact factors, investigating the occurrence mechanism of inconsistent phenomena, and counting the influence rates of these factors.

**Figure 1:**
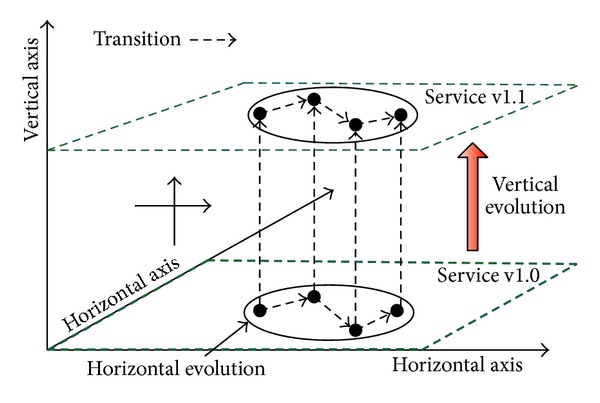
Three-dimensional view of service evolution.

In this paper, according to the evolution direction of services, the service consistency evolution can be divided into two categories: horizontal and vertical. Horizontal evolution refers to the internal consistency state changes for the same version of services and vertical evolution refers to service consistency problems between the same service's different versions. For each single service, we can describe its evolution processes by a three-dimensional view as shown in Figure 1. Additionally, the events of service evolution also can be summarized into two types: essential (ES) and nonessential (NES), on the basis of the product life cycle of service. The ES-events include service composition (SC), interface description (ID), Web encapsulation (WE), and version management (VM), referring to the processes of service's production and maintenance, whereas the NES-events such as redundant storage (RS), message interaction (MI), search and matching (SM), and share and reusage (SR) refer to the service's application processes. These events are the important elements of services' consistency state evolution.  Table 1 shows the detailed events or factors of service consistency evolution.

### 3.2. Inconsistency Factors

In this section some formal method-based concepts for modeling are first introduced. A service consistency evolution model EHS-FSA is then proposed based on FSA. The representation of Section 3.1 indicates that the consistency state of service evolution can be analyzed from macroscopical factors based on structures, behaviors, and functions of service, which consist of service interface protocols (*structure*), storage deployment (*structure*), service production and maintenance (*behavior*), message communication (*behavior*), share and reusage (*function*), matching between service publishers and subscribers (*function*), and other ones. For example, the failures of message interactions among different services can prevent service running due to the network delay, package loss, hardware or software troubles, different service may have a diversity of description styles such as HTTP, SOAP, WSDL, and XML [24], which makes services' calls and compositions more difficult.

In fact, service composition is very significant in SODSA. It means that one single service can be composed of different atomic services, in which the atomic service is function-simple and relatively independent, but this combinative service has a larger granularity and more applications [25]. The composition among different services can provide some value-added functions and meet the subscribers' requirements [25]. However, owing to the heterogeneous, distributed, and dynamic network environment, the service composition is affected by changes in communication mode, network block, denial attacks of service, infrastructure failures, and other issues [24], making the compositional behaviors of service not easy.

Note that the occurrence of any inconsistency factors in software engineering must have certain statistical regularity and these factors causing service inconsistency are no exception. Exactly speaking, the factors' importance degree should have a certain ordinal and numerical relationship, for instance, which factor is the least influential factor?, which factor is the most influential?, and what are the impact proportions of these factors?, respectively. Our main target in this paper is solving these problems by using formal method and FSA theory.

## 4. Consistency Evolution Model

In this section some formal method-based concepts for modeling are first introduced. A service consistency evolution model EHS-FSA is then proposed based on FSA.

### 4.1. Definitions and Notations

We first define related concepts which are the background knowledge of proposed EHS-FSA method and its algorithm for factors quantification analysis.

**Definition 1 (atomic service)**

We define AtS = {*α*_*i*_ | *i* ∈ N*} as the set of atomic services. *α*_*i*_ (abbreviated as *α*) is the smallest service unit in SODSA, which encapsulates several models into one kind of web service and can only complete a simple task.

**Definition 2 (composite service)**

Several atomic services can be appropriately assembled into a larger granular service. Let CoS = {*β*_*i*_ | *i* ∈ N*, *β*_*i*_ = 〈*α*_1_, *α*_2_, …, *α*_*k*_〉} denote the set of compositional services where *β*_*i*_ (abbreviated as *β*) is a *k*-tuple meaning the composite array and *k* denotes the number of *α*.

We assume that a single *α* that can be used to establish many *β* is the basic service unit and some service activities such as publishing, accessing, and calling can be executed only after the happen of *α*'s composition behavior.

**Definition 3 (global service space)**

We define a logically transparent space GSS = AtS ∪ CoS which consists of all *α* and *β* in SODSA system. For GSS, *α* and *β* in different physical nodes logically belong to the same node. That is, the service is transparent and seems to be deployed locally, although actually saved at geographically dispersed regions.

According to Definition 3, GSS is like a container in which each service interaction can be done locally and there donot exist the remote accesses and calls, and all service-related consistency state evolutions can be executed in GSS.

Assume that *β* can no longer be integrated into a higher level of service with other *β* after several *α* are assembled into it. That is, there are only two types of service in SODSA: *α* and *β*, indicating the number of service layer is 2.

**Figure 2:**
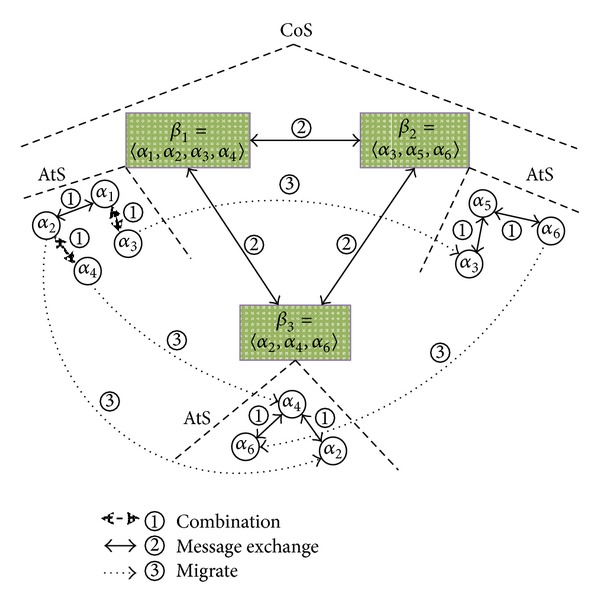
Example of service composition, reusage, and messages exchanges.

Figure 2 shows a simple example of *α*'s behaviors of composition and reusage and *β*'s message exchanges in GSS. In Figure 2, symbol “*①*” denotes the combination of different *α*, symbol “*②*” denotes message interactions between two business-related *β*, and symbol “*③*” denotes the migration of *α*, meaning the atomic services' share and reusage.

To simplify the problems and easily achieve formal descriptions, we consider only dominating influential events (or factors) on consistency state of services. These events include SC, WE, ID, SR, MI, and VM (as shown in Table 1).

**Definition 4 (consistency attributes of AtS)**

Let a three-tuple ATO_*α*_ = 〈VM_*α*_, WE_*α*_, SR_*α*_〉 denote the consistency-related properties set of *α* in GSS. The elements in ATO_*α*_ correspond to the before-mentioned events VM, WE, and SR, respectively, which should be carefully considered in maintaining the consistency state of *α*.

**Definition 5 (consistency attributes of CoS)**

In GSS, use a four-tuple COM_*β*_ = 〈ID_*β*_, MI_*β*_, SC_*β*_, Aset〉 to denote *β*'s consistency attributes set, where Aset = ATO_*α*_1__ ∪ ATO_*α*_2__ ∪ ⋯∪ATO_*α*_*k*__, *k* is the number of current *α* in corresponding *β*, ID_*β*_ denotes the factor ID's consistency state, MI_*β*_ represents the factor MI's correctness, SC_*β*_ denotes the composition capability of the factor SC.

Seen as Definition 4, we can observe that all attributes of *α* are included in relevant *β* attributes but are just a subset of COM_*β*_. Therefore, the consistency attributes of *β* can be divided into two levels: atomic layer and compositional layer. There are multiple *α* and *β* in GSS.

**Definition 6 (consistency state array of AtS)**

For all *t*, use the state array AtS_*α*_^*t*^ = (vm_*α*_, we_*α*_, *sr*⁡_*α*_) (abbreviated as AtS_*α*_^*t*^) to denote the monitoring values of atomic service *α*'s consistency status where *t* is the simulation time, the elements vm_*α*_, we_*α*_, and sr_*α*_ whose range is {true, false}, respectively, mapped the events VM, WE, and SR in ATO_*α*_. The logic term “true” denotes consistency, whereas “false” denotes inconsistency.

**Definition 7 (consistency state array of CoS)**

Let the composited state array CoS_*β*_^*t*^ = 〈(id_*β*_, mi_*β*_, sc_*β*_), AtS_*α*_^*t*^〉 (abbreviated as CoS_*β*_^*t*^) denote *β*'s monitor value of consistency states at any *t* where (id_*β*_, mi_*β*_, sc_*β*_) is called the root array, and AtS_*α*_^*t*^ is called the leaf array. The monitor values in CoS_*β*_^*t*^ whose range is uniform to that in Definition 6 mapped the attributes in COM_*β*_, respectively.

According to Definitions 6 and 7, AtS_*α*_^*t*^ and CoS_*β*_^*t*^ can quantitatively describe the consistency states of services. In the running of SODSA, we can use them for each *α* and *β* to monitor service state changes. Note that each *α* and *β* in GSS have eight such state arrays (2^3^ = 8).

Give an example of consistency state arrays: at *t*, assume that the state arrays of *α* are AtS_*α*_1__^*t*^ = (0,1, 1), AtS_*α*_2__^*t*^ = (1,1, 1), AtS_*α*_3__^*t*^ = (1,1, 0) and AtS_*α*_4__^*t*^ = (1,0, 0). If *α*_1_ is integrated with *α*_2_ to produce *β*_1_ and *β*_2_ is composed of *α*_3_ and *α*_4_, then we have the state arrays of *β* CoS_*β*_1__^*t*^ = 〈(1,0, 1), (0,1, 1)∪(1,1, 1)〉 and CoS_*β*_2__^*t*^ = 〈(1,1, 0), (1,1, 0)∪(1,0, 0)〉.

**Definition 8 (input events set of service states)**

Considering several primary consistency factors of *α* and *β*, we define the input events set of service states *I* = *AI* ∪ *CI*, *AI* = {*a*_*i*_ | *i* = 1,2,…, 6}, and *CI* = {*c*_*i*_ | *i* = 1,2,…, 6} where *AI* and *CI* are, respectively, the input set of state transition of *α* and *β*. The element *a*_*i*_ in *AI* refers to the following factors: version management confusion (*a*_1_), encapsulation troubles (*a*_2_), hard reusability (*a*_3_), version maintenance (*a*_4_), encapsulation repair (*a*_5_), and reusability improvement (*a*_6_). Similarly, *c*_*i*_ refers to these factors: different interface description (*c*_1_), message communication failure (*c*_2_), *α*'s noncomposition (*c*_3_), standardization of interface definition (*c*_4_), successful message transmission (*c*_5_), and *α*'s composability amendment (*c*_6_).

**Definition 9 (output events set of service states)**

The consistency state transition of services can be triggered by input events, resulting in the output events whose set is denoted by *O* = *AO* ∪ *CO*, *AO* = {*d*_*i*_ | *i* = 1,2,…, 6}, *CO* = {*h*_*i*_ | *i* = 1,2,…, 6}. *AO* and *CO* are the output set of state transition of *α* and *β*, respectively. For each *d*_*i*_, we define the following output events of *α*: version inconsistency (*d*_1_), *α*'s internal inconsistency (*d*_2_), nonreusability (*d*_3_), version consistency (*d*_4_), encapsulation correctness (*d*_5_), good reusability (*d*_6_). For each *h*_*i*_, involving the following *β*'s output events: inconsistent interface (*h*_1_), message exchanges inconsistency (*h*_2_), composition inconsistency (*h*_3_), interface consistency (*h*_4_), message interaction consistency (*h*_5_), and composition consistency (*h*_6_).

### 4.2. Extended Hierarchical Service-Finite State Automata

In this section, we construct an extended and hierarchical FSA to portray the dynamic evolution mechanism of service consistency states in SODSA.

**Definition 10 (extended hierarchical service-finite state automata, EHS-FSA)**

We define service consistency evolution as a set of extended FSA, which is formally denoted by *AC* = {*AC*_1_, *AC*_2_,…, *AC*_*i*_,…, *AC*_*m*_}. *AC*_*i*_ is an extended FSA represented by the nine-tuple AC⁡_*i*_ = 〈*L*, *L*_0_, *Q*, *Q*_0_, *I*, *O*, *TS*, *δ*, *λ*〉, where:

- *L* = {AtS_*α*_^*t*^ | *i* = 1,2,…, *k*} and *Q* = {CoS_*β*_^*t*^ | *i* = 1,2,…, *k*} denote the finite and nonempty set of consistency states of *α* and *β* in *AC*_*i*_, respectively,
- *L*_0_ ⊂ *L* and *Q*_0_ ⊂ *Q* are the sets of all state arrays of initial *α* and *β*, respectively,
- *I* and *O* denote the finite and nonempty set of service state transition's input events and output ones, respectively,
- $\text{TS}=\left\{\text{ts}|\text{ts}=(\text{Co}{\text{S}}_{\beta_{j}}^{t},\text{At}{\text{S}}_{\alpha }^{t})\overset{I/O}{\to }(\text{Co}{\text{S}}_{\beta_{j}}^{t^{\prime }},\text{At}{\text{S}}_{\alpha }^{t^{\prime }})\right\}$ represents the rule set of state transition of service consistency, that is the changes of state arrays CoS_*β*_*j*__^*t*^ and AtS_*α*_^*t*^ triggered by *I* from the interval *t* to *t*′,
- *δ* : (*Q*, *L*) × *I* → (*Q*, *L*) is the state transition function of service evolution,
- *λ* : (*Q*, *L*) × *I* → *O* is the output event function.

In EHS-FSA, we have such transition functions *δ*(AtS_*α*_^*t*^, *AI*) = AtS_*α*_^*t*′^, *δ*(CoS_*β*_^*t*^, CI) = CoS_*β*_^*t*′^, and so on. To simplify the transition rule, we assume that there only exists one input event in *I* and one output event in *O* for each state transition. According to Definition 10, there are partial state transition details for each *α* as shown in Table 2.

**Figure 3:**
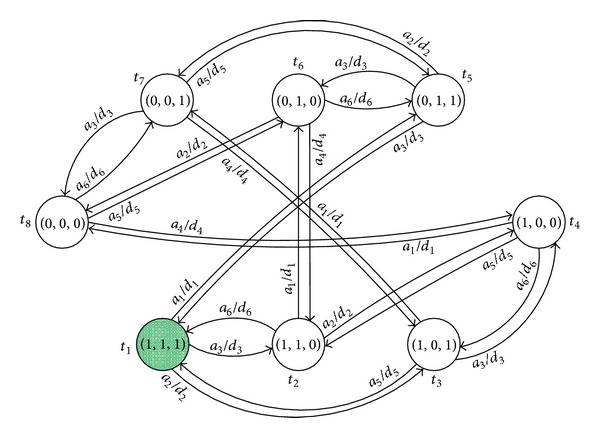
State transition view of *α*.

Figure 3 gives an illustrated way to portray *α*'s state transition processes, in which the rule *a*_*i*_/*d*_*i*_ denotes the input event and output event for each *α*'s state change, *t*_*i*_ is the current system time, and the symbol “$\overset{a_{i}/d_{i}}{\to }$” represents the service state's evolution behavior. For example, the state array (0,0, 0) evolves into (0,0, 1) triggered by the rule *a*_6_/*d*_6_. Similarly, there are the similar state transition courses of root arrays in *β*.

**Definition 11 (states transition matrix of *α*)**

We define the following transition matrix:

(1) $$\begin{array}{c} \Pi ={\left[\text{At}{\text{S}}_{\alpha }^{t}\right]}_{m\times n}, \end{array}$$

where AtS_*α*_^*t*′^ = *δ*(AtS_*α*_^*t*^, *a*_*i*_), *a*_*i*_ ∈ *AI* (*i* = 1,2,…, *n*), *m* denotes the number of all *α* states, and *n* denotes the amount of *α*'s state input events that are listed by the sequence in *AI*. If the transition function *δ*(AtS_*α*_^*t*^, *a*_*i*_) does not exist, AtS_*α*_^*t*^ in Π is represented by 0; contrarily, the state arrays (1,1, 1), (1,1, 0), (1,0, 1), (1,0, 0), (0,1, 1), (0,1, 0), (0,0, 1) and (0,0, 0) are, respectively, denoted by the values 1, 2, 3, 4, 5, 6, 7, and 8 in Π.

We can find that the number of *α*'s actual states is 8 and the number of *AI* events is 6. Hence, the actual Π can be obtained as follows:

(2) $$\begin{array}{c} \prod =\left[\begin{array}{cccccc} 5 & 3 & 2 & 0 & 0 & 0\\ 6 & 4 & 0 & 0 & 0 & 1\\ 7 & 0 & 4 & 0 & 1 & 0\\ 8 & 0 & 0 & 0 & 2 & 3\\ 1 & 7 & 6 & 0 & 0 & 0\\ 0 & 8 & 0 & 2 & 0 & 5\\ 0 & 0 & 8 & 3 & 5 & 0\\ 0 & 0 & 0 & 4 & 6 & 7 \end{array}\right]. \end{array}$$

It can be observed that the contents of matrix Π are consistent with those of *α* in Table 2. Similarly, we can define the state transition matrix of *β*'s root arrays as *Z* = [CoS_*β*_^*t*^]_*m*×*n*_.

**Definition 12 (occurrence probability of event set *I*)**

We use *P*(*a*_*i*_) and *P*(*c*_*i*_) to denote the occurrence probability of events *a*_*i*_ and *c*_*i*_ in *I* respectively. In our model, assume that ∑*P*(*a*_*i*_) = 1, *P*(*a*_1_) = *P*(*a*_4_), *P*(*a*_2_) = *P*(*a*_5_) and *P*(*a*_3_) = *P*(*a*_6_); similarly, ∑*P*(*c*_*i*_) = 1, *P*(*c*_1_) = *P*(*c*_4_), *P*(*c*_2_) = *P*(*c*_5_), and *P*(*c*_3_) = *P*(*c*_6_).

In general, the majority of *α* and *β* are difficult to be reused and combined, due to the heterogeneity of distributed systems and the diversity of modeling methods and tools and the knowledge differences in different application fields in SODSA. Therefore, the events *a*_3_ and *c*_3_ will become the very frequent activities. In addition to developing some new services, the events *a*_2_ and *c*_1_ also will occur usually when fusing the functions and applications in previous and old simulation systems into the new SODSA. From the statistical point of view, the operation running of software systems is basically stable, so the events *a*_1_ and *c*_2_ will occur occasionally.

On the basis of the above-mentioned actual situations, we assume that the occurrence probabilities of *I* will satisfy the following constraints in the dynamic evolution processes of EHS-FSA: *P*(*a*_3_) > *P*(*a*_2_) > *P*(*a*_1_), *P*(*c*_3_) > *P*(*c*_2_) > *P*(*c*_1_), *P*(*a*_6_) > *P*(*a*_5_) > *P*(*a*_4_), and *P*(*c*_6_) > *P*(*c*_5_) > *P*(*c*_4_).

## 5. Service Consistency Evolution Algorithms

In this section, two algorithms SCEA-*α* and SCEA-*β* to monitor service consistency transition activities are constructed, which focus on the occurrence of impact factors in SODSA on the foundation of executing EHS-FSA. Both SCEAs achieve a dynamic analysis of the influencing factors that lead to inconsistency of *α* and *β*. By statistically counting the number of effects of each factor in the operation running of EHS-FSA, ultimately, the importance of factors can be calculated to get a quantitative analysis results.

At the theoretical level, both SCEAs are dynamic running of EHS-FSA. That is, the dynamic transition processes of service consistency states are modeled by them in software simulation environment with time advancement, in which the continually changing behaviors and their consistency states referring to the elements of EHS-FSA can be monitored. At the application level, SCEAs can identify the factors' dynamic behaviors that cause *α* and *β*'s inconsistency phenomena in SODSA and statistically draw the amount of each factor's occurrence to quantitatively analyze the evolution essence of service inconsistency.

The inputs of SCEA-*α* involve the following: (1) *α*'s amount *N* and the cycle number *K* are initialized. (2) The counting values of inconsistent factors are initialized: Num_VM_, Num_SE_, Num_SR_ ← 0 (refer to the events *a*_1_, *a*_2_, and *a*_3_, resp.). (3) Initialize *α*'s state arrays: AtS_*α*_*i*__^*t*^ ∈ *L*_0_ ← (1,1, 1). The outputs of SCEA-*α* include: the final numbers of factors occurrence that results in service inconsistency is determined: Num_VM_, Num_SE_, Num_SR_ and (i.e., the ultimate amounts of state transition events VM, SE, and SR). The detailed processes of SECA-*α* is described as the pseudocode in Algorithm 1.

The inputs of SCEA-*β* are as follows. (1) Initialize *β* and *α*'s counts *M*, *N*, and the cycle number *K*. (2) The original values of inconsistent factors' counting are assigned: Num_ID_, Num_MI_, Num_SC_ ← 0 (refers to the events *c*_1_, *c*_2_, and *c*_3_, resp.). (3) *α*'s state arrays are initialized: AtS_*α*_*i*__^*t*^ ∈ *L*_0_ ← (1,1, 1). (4) The initial composition schema of *α*: select several *α* randomly. (5) Initialize *β*'s state arrays: CoS_*β*_*j*__^*t*^ ∈ *Q*_0_ ← ((1,1, 1), AtS_*α*_*i*__^*t*^ ∪ AtS_*α*_*l*__^*t*^⋯). The outputs of SCEA-*β* are: the ultimate amounts of *β* inconsistency states' factors: Num_ID_, Num_MI_, and Num_SC_.  Algorithm 2 gives the pseudocode description of SECA-*β*'s detailed courses.

For the sake of the initial quantitative and dynamic exploration into service inconsistency factors and their evolution mechanism, therefore, ill-considered facets in SECAs design will inevitably appear, but the idea of using such novel analysis is an innovational and significative approach.

## 6. Quantitative Evaluation Experiment

### 6.1. Simulation Initialization

We implement the experimental evaluation using MATLAB R2009a on a PC with a Genuine Intel CPU T2400 (1.83 GHz) and 3.0 GB RAM, operated in Windows XP. We set the original number of *α* as 2–4 stochastically. Actually, owing to the small difference in the total number of *α* that minimally affects the algorithms' outputs when its count exceeds 50, the number of initial *α* could have been 5, 6, or 7 too. For example, if the total number of *α* is 156 or 157, the proportions of inconsistency factors (or events) remain at the same level when *α*'s count is 155 in simulation experiments.

At the initial evolution moment, several atomic services (*α*) are randomly composited into one *β*. The importance of each inconsistency factor (i.e., Num_VM_, Num_SE_, Num_SR_, Num_ID_, Num_MI_, and Num_SC_) can then be determined. To get the trustier results, we deploy four groups of experiments to execute the algorithms SECA-*α* and SECA-*β* where the total cycle numbers of them are 100, 200, 300, and 400. In addition, the selection of the input events in evolving EHS-FSA depends on the probability and constraints of the events occurrence defined in Section 4.2. For the exactly and credibly statistical analysis, all the results of the four experiments are averaged.

### 6.2. Results and Analysis

Table 3 shows that the experimental counting results on the effects of factors on service consistency which were derived by taking a different *K* and running the programs of SECA-*α* and SECA-*β*. According to the collected data in Table 3, the impact ratios of service inconsistency events can be manually calculated, as shown in Table 4, whose data are a more accurate revelation.

**Figure 4:**
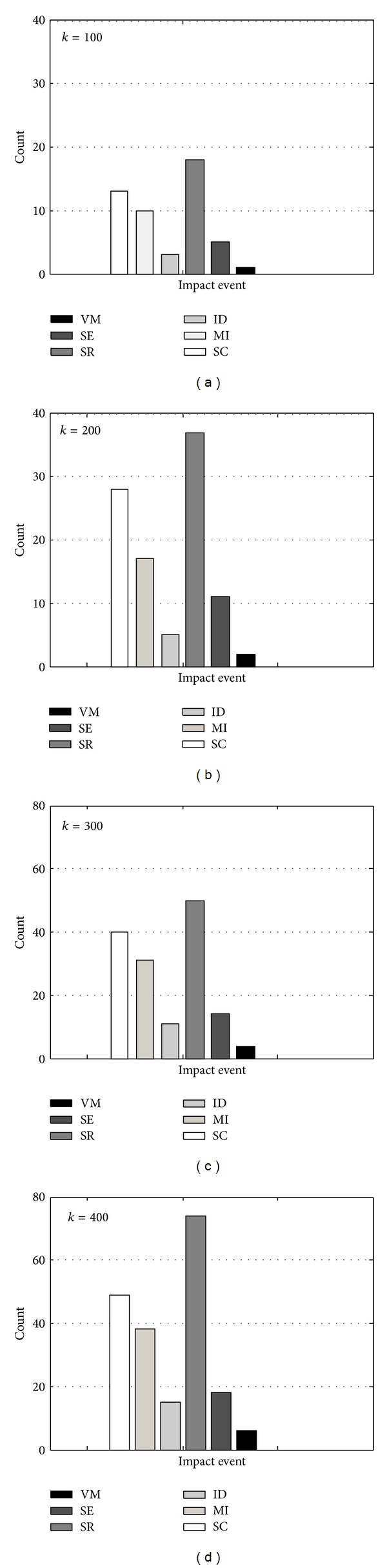
Comparison of the importance of different impact events.

**Figure 5:**
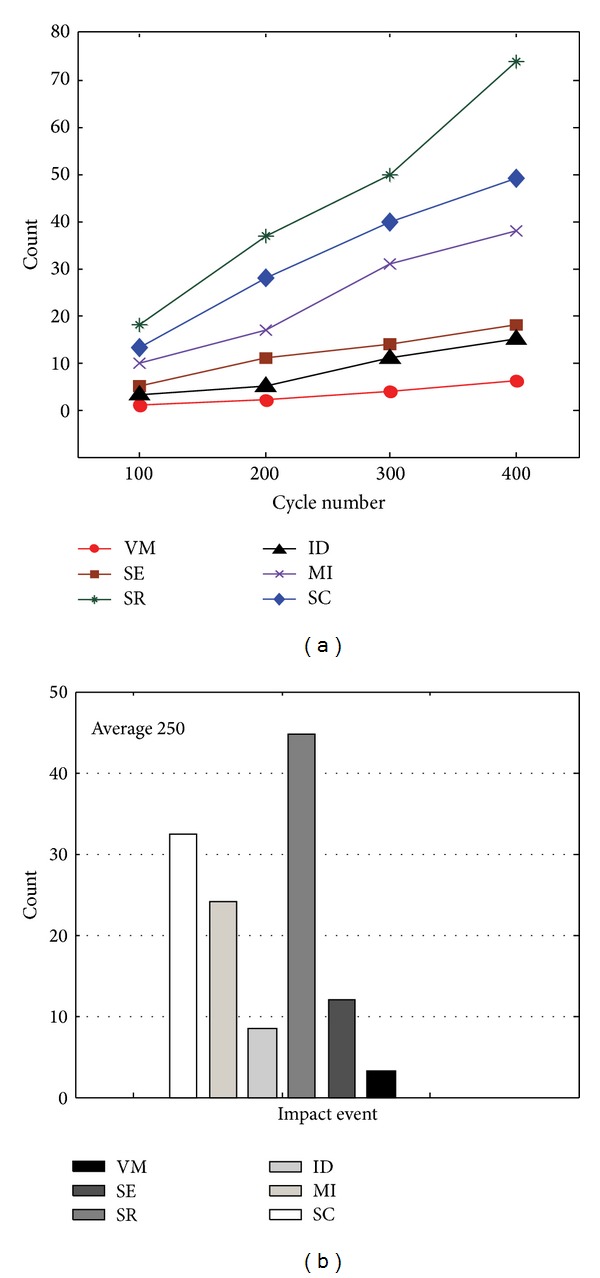
(a) Changing trends of factorial effects. (b) Average importance of factors at four cycle times.

To facilitate our experimental analysis in a more obvious manner, the statistical results in Table 3 are exhibited using the bar charts in Figures 4 and 5(b). Figures 4(a)–4(d) illustrate the continually rising times of each influence factor's occurrence while the algorithms' cycle number *K*, respectively equals 100, 200, 300, and 400, which imply the importance degree for service inconsistency, as shown in Table 4. It can be observed from Figure 4 and Table 4 that the order of these factors' importance degree is listed as follows.

1. *K* = 100: *a*_3_ (18%) > *c*_3_ (13%) > *c*_2_ (10%) > *a*_2_ (5%) > *c*_1_ (3%) > *a*_1_ (1%).
2. *K* = 200: *a*_3_ (18.5%) > *c*_3_ (14%) > *c*_2_ (8.5%) > *a*_2_ (5.5%) > *c*_1_ (2.5%) > *a*_1_ (1%).
3. *K* = 300: *a*_3_ (16.7%) > *c*_3_ (13.3%) > *c*_2_ (10.3%) > *a*_2_ (4.7%) > *c*_1_ (3.7%) > *a*_1_ (1.3%).
4. *K* = 400: *a*_3_ (18.5%) > *c*_3_ (12.25%) > *c*_2_ (9.5%) > *a*_2_ (4.5%) > *c*_1_ (3.75%) > *a*_1_ (1.5%).

From the listed-above sequences, we can observe that factor *a*_3_ is the biggest influential one leading to inconsistent states of atomic service *α* and factor *a*_1_ exerts minimal effect on *α*'s consistency states. These sequences also indicate that the order of influencing proportion at different cycle times of SECA-*α* and SECA-*β* is the same despite the ratios' tiny differences.

In the following parts, we discussed what and why are the actual situations of the influencing rates of factors on service consistency evolution, as indicated by the experiment outcomes in Tables 3 and 4 and Figure 4. We combined some actual software development examples related to distributed service deployment in local area network (LAN) or wide area network (WAN) and evaluations results in this paper to discover some regular behaviors and give significative guidance for actual service consistency maintenance.

(1) The factors with very high impact (ranked 1 and 2) are *α*'s bad reusability (*a*_3_) and *α*'s noncomposition (i.e., several atomic services are not composited into one larger service *β*) (*c*_3_). This is mainly for the reasons: firstly, there is a great deal of incompatibilities in interfaces and deployment styles as fusing the old distributed simulation systems into the current SODSA; secondly, there exist many different development tools such as C++, JAVA, MATLAB, Simulink, LABVIEW, and so forth; thirdly, the produced services are from different modeling domains such as electrics, communication system, machine design, hardware, and software. Such heterogeneity and diversity are prevalent, increasing the difficulty of service combination and reusability. These situations are based on objective facts in SODSA deployment and are not easy to be changed nowadays.

(2) The third important factor (ranked 3) is *β*'s message communication failure (*c*_2_). From the perspective of service application layer, we can say that *c*_2_ is actually the most important influential factor on services inconsistency because in current or future SODSA, *message* is the most popular medium for service communication, and the message interactions between services are frequent and also can be easily blocked, due to the services' enormous amount, the continually entry and exit of simulation nodes, the instability of distributed network, and so on.

(3) The fourth and fifth factors with moderate impact (ranked 4 and 5) are *α*'s encapsulation failure (*a*_2_) and *β*'s interface description difference (*c*_1_), respectively. This is mainly because *α*'s encapsulation and *β*'s interface description are the basis of service calls, which occupy an important position in simulation service developments and directly affect such services behaviors like composition, reusage, interaction, and so on.

(4) The confusion of version management (*a*_1_) is the smallest influential factor (ranked 6). In general, the management mechanism of simulation services is usually effective and stable once it has been established.

Figure 5(a) indicates that the impact of each factor on service inconsistency states is continuously strengthened with the increasing cycle number *K* in SECA. We can also observe from Figure 5(a) that event *a*_3_ has the greatest increasing effect on *α*, events *c*_3_ and *c*_2_ exert a greater increasing influence on *β*, whereas events *a*_2_ and *c*_1_ have a very small changing extent to affect *α* and *β*'s consistency states, respectively, and *a*_1_ has the smoothest changes whose effect on *α* is the smallest. On the basis of the average statistics at four cycle times, Figure 5(b) depicts the influencing factors' bar comparison by which the sorting of these factors and their impact rate is *a*_3_ (17.93%) > *c*_3_ (13.12%) > *c*_2_ (9.58%) > *a*_2_ (4.93%) > *c*_1_ (3.24%) > *a*_1_ (1.2%). Obviously, this order is consistent with the results in Figure 4 and each factor's impact rates at different cycle times only have a tiny difference, which improve the reliability of our experiment.

The above analysis results can provide an important concern on overcoming the service inconsistency risks in the design, development, publishing and subscription, operation running and maintenance of services in SODSA. To say the least, our experimental results may not completely reflect the actual influencing mechanism of service inconsistency factors, but most of them are in line with the actual situation. After all, in service evolution processes, the behaviors are complex, the attributes are various, and the system size is unpredictable, and so on. Hence, we must make some reasonable assumptions and self-defined rules by which the simulation evaluation can be finished effectively.

## 7. Conclusion

In this paper we propose an extended hierarchical finite state automata EHS-FSA and its two subalgorithms (SCEA-*α* and SCEA-*β*) to monitor the consistency states changes of *α* and *β* in SODSA. Based on theoretical and macroscopic perspective, EHS-FSA can formally portray the dynamic impact mechanism of service inconsistency behaviors and attributes in terms of reusability, composition, message exchange, service encapsulation, and so on. The presented SCEA aims to achieve a quantitative analysis of the inconsistency factors in service evolution for compositional SODSA, by running the EHS-FSA automata.

This study represents our preliminary attempt to introduce a novel analysis method of inconsistency factors which is completely different from previous ones. Our quantitative evaluation experiments show that EHS-FSA and SCEA are feasible, effective, advanced, and superior to traditional ones. The research achievements can offer theoretical and technical guidance for reducing service inconsistency states and improving the correctness of simulation services. In addition, our methods also can be applied to other domains such as the analysis and design of distributed-cooperative command posts that are deployed in future battlefields, complex distributed information systems based on network-centric warfare, embedded real-time systems.

Some future researches are as follows: (1) more inconsistency factors should be focused. (2) Some in-depth analysis of the disturbance mechanism among factors should be done. (3) The probability of event set *I* should be considered more carefully. (4) Related maintenance technologies of service consistency can be developed based on our experiment results.

## Figures and Tables

**Algorithm 1:**
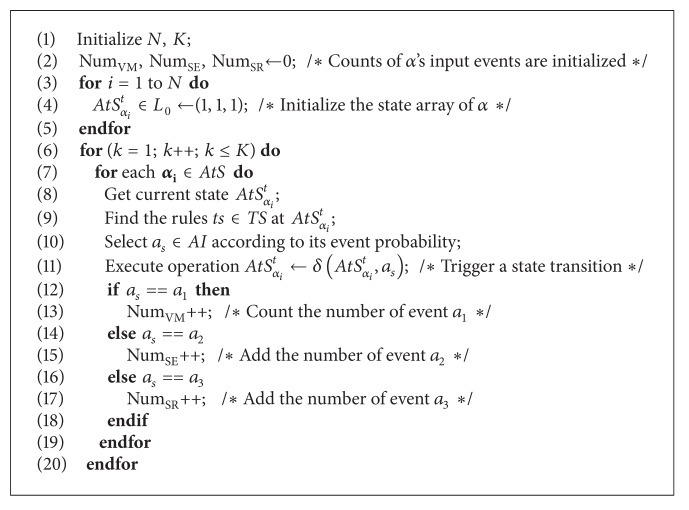
Pseudocode of SCEA-*α* algorithm.

**Algorithm 2:**
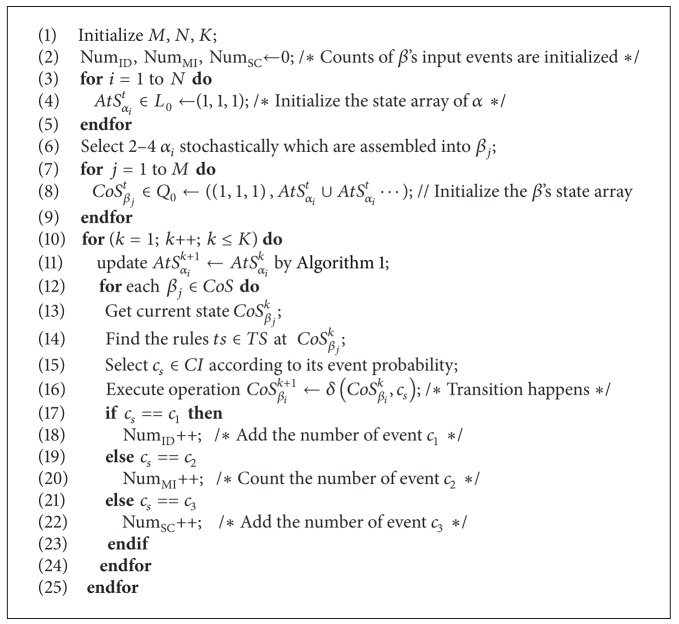
Pseudocode of SCEA-*β* algorithm.

**Table 1:** Inconsistency influencing events and their categorization.

Event	Type	Consistency meaning
SC	ES	Feasibility and integrity
ID	ES	Diversity of interface description
RS	NES	maintenance of several backups for the same service
WE	ES	Encapsulation correctness of web service based on models
VM	ES	Version control for the same service
MI	NES	Service communication without failure
SM	NES	Services' matching degree between publishers and subscribers
SR	NES	Whether the same service can be called by multiple other services

**Table 2:** Transition lists of *α*'s partial consistency states.

Current state	*I*	*δ*	*O*	Next state
(1, 1, 1)	*a*_1_	*δ* ((1, 1, 1), *a*_1_)	*d*_1_	(0, 1, 1)
(1, 1, 1)	*a*_3_	*δ* ((1, 1, 1), *a*_3_)	*d*_3_	(1, 1, 0)
(1, 1, 1)	*a*_2_	*δ* ((1, 1, 1), *a*_2_)	*d*_2_	(1, 0, 1)
(1, 1, 0)	*a*_1_	*δ* ((1, 1, 0), *a*_1_)	*d*_1_	(0, 1, 0)
(1, 1, 0)	*a*_6_	*δ* ((1, 1, 0), *a*_6_)	*d*_6_	(1, 1, 1)
(1, 1, 0)	*a*_2_	*δ* ((1, 1, 0), *a*_2_)	*d*_2_	(1, 0, 0)
(1, 0, 1)	*a*_1_	*δ* ((1, 0, 1), *a*_1_)	*d*_1_	(0, 0, 1)
(1, 0, 1)	*a*_3_	*δ* ((1, 0, 1), *a*_3_)	*d*_3_	(1, 0, 0)
(1, 0, 1)	*a*_ 5_	*δ* ((1, 0, 1), *a*_5_)	*d*_5_	(1, 1, 1)
(1, 0, 0)	*a*_1_	*δ* ((1, 0, 0), *a*_1_)	*d*_1_	(0, 0, 0)
(1, 0, 0)	*a*_5_	*δ* ((1, 0, 0), *a*_5_)	*d*_5_	(1, 1, 0)
(1, 0, 0)	*a*_ 6_	*δ* ((1, 0, 0), *a*_6_)	*d*_6_	(1, 0, 1)

**Table 3:** The number of factors occurrences affecting service inconsistency.

*K*	*a*_1_	*a*_2_	*a*_3_	*c*_1_	*c*_2_	*c*_3_
100	1	5	18	3	10	13
200	2	11	37	5	17	28
300	4	14	50	11	31	40
400	6	18	74	15	38	49

Average (250)	3.25	12	44.75	8.5	24	32.5

**Table 4:** The impact rates of service inconsistency factors.

*K*	*a*_1_	*a*_2_	*a*_3_	*c*_1_	*c*_2_	*c*_3_
100	1%	5%	18%	3%	10%	13%
200	1%	5.5%	18.5%	2.5%	8.5%	14%
300	1.3%	4.7%	16.7%	3.7%	10.3%	13.3%
400	1.5%	4.5%	18.5	3.75%	9.5%	12.25%

Average (250)	1.2%	4.93%	17.93%	3.24%	9.58%	13.12%
